# Mitotic Inheritance of PRC2-Mediated Silencing: Mechanistic Insights and Developmental Perspectives

**DOI:** 10.3389/fpls.2020.00262

**Published:** 2020-03-09

**Authors:** Alice Hugues, Chean Sern Jacobs, François Roudier

**Affiliations:** ^1^Laboratoire Reproduction et Développement des Plantes, ENS de Lyon, UCB Lyon 1, CNRS, INRAE, INRIA, Université de Lyon, Lyon, France; ^2^Master de Biologie, École Normale Supérieure de Lyon, Université Claude Bernard Lyon I, Université de Lyon, Lyon, France

**Keywords:** polycomb repressive complex 2, H3K27me3 inheritance, epigenetic memory, chromatin, replication

## Abstract

Maintenance of gene repression by Polycomb Repressive Complex 2 (PRC2) that catalyzes the trimethylation of histone H3 at lysine 27 (H3K27me3) is integral to the orchestration of developmental programs in most multicellular eukaryotes. Faithful inheritance of H3K27me3 patterns across replication ensures the stability of PRC2-mediated transcriptional silencing over cell generations, thereby safeguarding cellular identities. In this review, we discuss the molecular and mechanistic principles that underlie H3K27me3 restoration after the passage of the replication fork, considering recent advances in different model systems. In particular, we aim at emphasizing parallels and differences between plants and other organisms, focusing on the recycling of parental histones and the replenishment of H3K27me3 patterns post-replication thanks to the remarkable properties of the PRC2 complex. We then discuss the necessity for fine-tuning this genuine epigenetic memory system so as to allow for cell fate and developmental transitions. We highlight recent insights showing that genome-wide destabilization of the H3K27me3 landscape during chromatin replication participates in achieving this flexible stability and provides a window of opportunity for subtle transcriptional reprogramming.

## Introduction

Polycomb repressive complex 2 (PRC2) is a conserved chromatin-modifying complex that catalyzes the trimethylation of lysine 27 of histone H3 (H3K27me3) (Margueron and Reinberg, 2011; Schuettengruber et al., 2017). PRC2 is composed of four core subunits that are necessary for its histone methyltransferase activity: Nurf55, suppressor of zeste 12 [Su(z)12], extra sex combs (ESC), and enhancer of zeste [E(z)], as originally identified in Drosophila. In multicellular organisms, members of these core subunits are often present in multigene families. For instance in the flowering plant *Arabidopsis thaliana*, the Su(z)12 and E(z) subunits are encoded by three homologous genes, leading to several PRC2 complexes with potentially distinct biochemical properties and developmental roles (Mozgova and Hennig, 2015). Further functional diversity is brought about by an array of additional factors that direct PRC2 recruitment to specific loci or affect the activity of the complex (Yu et al., 2019).

Polycomb repressive complex 2 activity orchestrates developmental and cellular programs by preserving the integrity of the gene expression patterns that underpin cell identity and function. Genetic and molecular evidence obtained from many organisms indicate that PRC2 activity is not required to initiate transcriptional repression but is necessary to maintain target gene repression, thereby providing a cellular memory system during development (Schuettengruber et al., 2017; Reinberg and Vales, 2018). Two remarkable properties lie at the heart of this genuine epigenetic process. First, the coupling between PRC2 *writing* and *reading* activities enables H3K27me3 self-propagation over large chromatin domains from an initially small number of nucleating nucleosomes marked by H3K27me3 (Oksuz et al., 2018; Yu et al., 2019). Second, H3K27me3 patterns are faithfully inherited from mother to daughter cells despite chromatin disassembly ahead of the replication fork that directly conflicts with the transmission of histone post-translational modifications (PTMs) to daughter cells (Annunziato, 2015; Masai and Foiani, 2018). The discoveries that parental histones are recycled and reincorporated into nascent chromatin and that H3K27me3 levels are restored downstream of the replication fork in both animal and plant cell cultures (Xu et al., 2012; Alabert et al., 2015; Jiang and Berger, 2017) highlight the fact that the S-phase is not only about replicating DNA, but also chromatin together with its epigenetic potential (Ramachandran et al., 2017; Escobar et al., 2018; Reverón-Gómez et al., 2018; Serra-Cardona and Zhang, 2018).

The molecular mechanisms responsible for the faithful perpetuation of H3K27me3-marked chromatin through cell division are under active investigation. Whereas strong evidence indicates that H3K27me3 itself is the physical support of the PRC2-based memory system (Xu et al., 2012; Coleman and Struhl, 2017; Laprell et al., 2017), it might not be the only carrier of this epigenetic process *in vivo* (Højfeldt et al., 2018; Sharif and Koseki, 2018). The first part of this review aims at presenting current understanding of the histone recycling machinery and of the self-perpetuation properties that underlie the inheritance of H3K27me3 in nascent chromatin. We then discuss the fact that, in addition to its remarkable stability, this memory system also needs to be flexible and that chromatin replication likely provides a window of opportunity enabling the transcriptional changes that drive cell fate decisions and developmental transitions.

## Molecular Mechanisms Underlying the Mitotic Inheritance of PRC2-Mediated Repression

### Recycling of H3K27me3-Marked Nucleosomes and Incorporation of Neo-Synthesized Histones Into Nascent Chromatin

**FIGURE 1:**
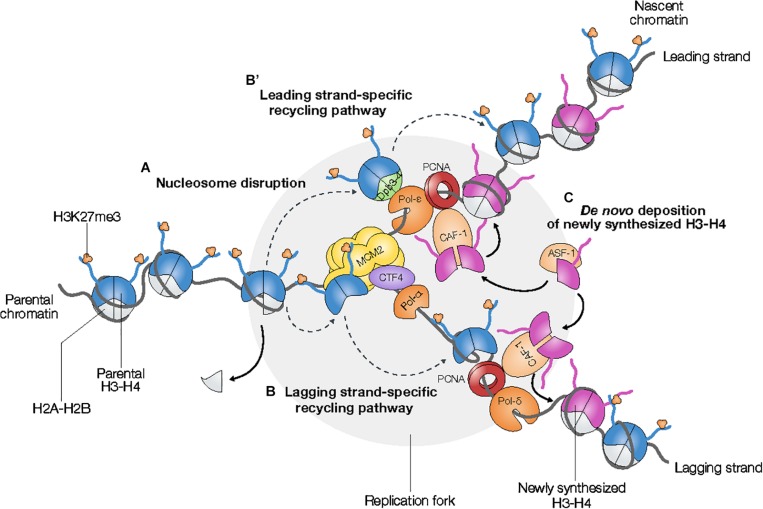
Parental K27-trimethylated histone H3 are transmitted to nascent chromatin via the recycling of histones H3 at the replication fork. **(A)** Parental nucleosomes are disrupted ahead of the replication fork. The putative mechanisms underlying the recycling of parental H3s into nascent chromatin are strand-dependent (**B,B′** dashed gray arrows). Parental H3s are guided either to the leading strand **(B′)** or to the lagging strand **(B)** *via* stand-specific pathways involving the heterodimer Dpb3-4 associated to Pol-ϵ and the MCM2-CTF4-Pol-α axis, respectively. **(C)** *De novo* incorporation of newly synthesized H3–H4 dimers *via* histone chaperones ASF-1 and CAF-1 leads to the twofold dilution of parental H3K27me3 levels in nascent chromatin.

Parental nucleosomes disassembly at the replication fork (Teves and Henikoff, 2014; Annunziato, 2015; Masai and Foiani, 2018) is at odds with the perpetuation of parental H3K27me3 patterns (Figure 1A). In order to ensure that both daughter cells inherit the same parental epigenetic information, parental H3–H4 histones should be equiprobably distributed between the leading and the lagging strands downstream of the replication fork. However, the structural asymmetry of the replication fork is likely to induce a bias during the re-deposition of parental histones into nascent chromatin (Snedeker et al., 2017). Numerous studies in yeast and mammalian cells showed that cells manage to compensate for this intrinsic asymmetry via the intricate cooperation between histone chaperones and the DNA replication machinery that enables accurate recycling of parental histones together with their epigenetic marks (Hammond et al., 2017).

Recent studies uncovered strand-specific pathways of parental histone recycling in nascent chromatin (He et al., 2017; Gan et al., 2018; Petryk et al., 2018; Yu et al., 2018). The transfer of parental histones H3–H4 to the lagging strand relies on the synergistic action of the histone chaperone Mini-Chromosome Maintenance Protein 2 (MCM2), a subunit of the MCM helicase, chromosome transmission fidelity 4 (CTF4), and the lagging strand-specific primase DNA polymerase α (Pol-α) (Huang et al., 2015; Gan et al., 2018; Petryk et al., 2018; Figure 1B). CTF4 anchors Pol-α in the vicinity of the MCM helicase, thus enabling the transfer of parental histones H3–H4 from MCM2 to the lagging stand via Pol-α. The transfer of parental histones H3–H4 to the leading strand depends on Dpb3-4, a heterodimer associated with the leading strand-specific DNA polymerase ϵ (Pol-ϵ) (He et al., 2017; Yu et al., 2018; Figure 1B′). Interestingly, asymmetric inheritance of parental histones H3–H4 in fission yeast lacking either Dpb3 or Dpb4 results in loss of heterochromatin integrity and transcriptional activation, which emphasizes the functional role of epigenetic inheritance in the maintenance of genome stability and transcriptional programs (He et al., 2017; Yu et al., 2018).

Incorporation of newly synthesized H3–H4 dimers into nascent chromatin involves the two histone chaperones anti-silencing function protein 1 (ASF-1) and chromatin assembly factor 1 (CAF-1) (Hammond et al., 2017; Figure 1C). Since ASF-1 co-binds parental H3–H4 histones together with MCM2, nucleosome assembly of neo-synthesized histones driven by ASF-1 and CAF-1 might be also involved in the re-deposition of parental histones H3–H4 into nascent chromatin (Huang et al., 2015). Whereas the role of the Arabidopsis ASF-1 homologs AtASF1a/b remains unclear, CAF-1-dependent incorporation of newly synthesized histones is important for the efficient maintenance of histone PTM levels during replication (Jiang and Berger, 2017; Benoit et al., 2019).

Despite its efficiency, the recycling of parental H3 into nascent chromatin is not sufficient on its own to enable the full restoration of H3K27me3 in daughter cells. Indeed, parental H3K27me3 level is diluted twofold after chromatin replication due to the incorporation of newly synthesized, unmethylated histones H3–H4 into nascent chromatin that is required to re-establish the initial density of nucleosomes (Figure 1). Therefore, faithful transmission of PRC2-mediated gene repression downstream of the replication fork requires specific mechanisms to spread H3K27me3 from parentally modified to newly synthesized, unmodified H3 (Xu et al., 2012; Alabert et al., 2015; Jiang and Berger, 2017).

### Filling the Gaps? H3K27me3 Spreading Downstream of the Replication Fork

#### What Happens to PRC2 at the Passage of the Replication Fork?

**FIGURE 2:**
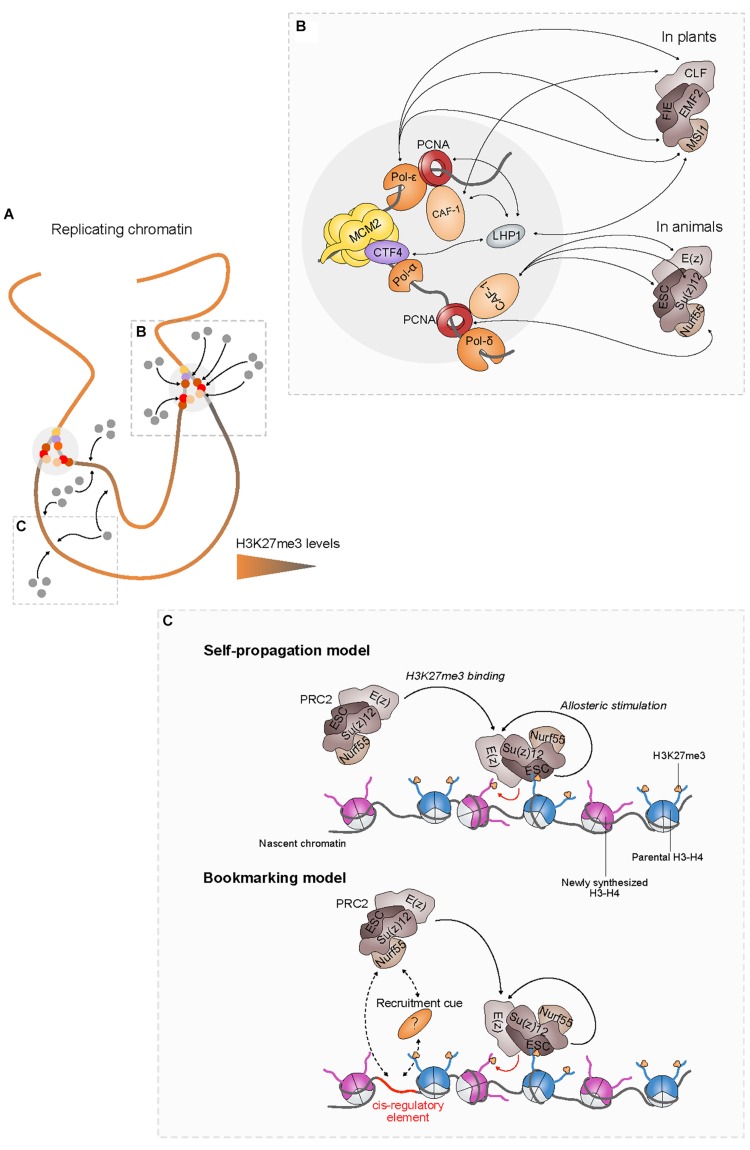
PRC2 recruitment at the replication fork and in nascent chromatin enables faithful restoration of the H3K27me3 landscape. **(A)** In replicating chromatin, H3K27me3 levels (represented by a colored gradient) are diluted twofold during replication. PRC2 (gray dots) recruitment at the fork via components of the replication machinery (colored dots) **(B)** and in nascent chromatin **(C)** facilitates the restoration of parental H3K27me3 levels. **(C, Top)** According to the self-propagation model, the positive feedback loop between PRC2 and H3K27me3 is sufficient to propagate H3K27me3 from inherited to newly synthesized H3, thereby ensuring the maintenance of PRC2-mediated transcriptional silencing through cell division. **(C, Bottom)** *Cis*-regulatory elements might also play a critical role for the restoration of H3K27me3 patterns by enhancing directly or indirectly the recruitment of PRC2 to nascent chromatin.

The passage of the replication fork does not only destabilize nucleosomes but also results in the eviction of most chromatin- and DNA-binding proteins, including PRC2. Nevertheless, pioneer studies based on cytological and *in vitro* approaches indicated that PRC2 remains localized around DNA replication sites (Hansen et al., 2008). In agreement with these observations, higher-resolution proteomic analyses showed that PRC2 is already associated to nascent chromatin immediately after the passage of the replication fork, suggesting that PRC2 is actively re-established over nascent chromatin thereby ensuring the spreading of H3K27me3 from parental to newly synthesized, unmethylated H3 (Alabert et al., 2014; Figure 2A).

##### The 3D organization of chromatin might facilitate local retention of PRC2 at the replication fork

H3K27me3-marked chromatin domains have been shown to form foci in the nucleus of mammalian cells (Oksuz et al., 2018). Similarly, in plant interphasic nuclei, PRC2 is enriched in nuclear speckles containing PWWP-DOMAIN INTERACTOR OF POLYCOMBS1 (PWO1), a factor that interacts with lamin-like proteins (Mikulski et al., 2019). Although their presence in replicating cells remains to be formally established, such PRC2-enriched micro-environments could participate in maintaining PRC2 close to its targets during the passage of the fork. At a larger scale, loss of nuclear compartmentalization in the Arabidopsis *3h1* mutant lacking histone H1 correlates with a global diminution in H3K27me3 occupancy. This indicates that the 3D organization of chromatin into subnuclear domains probably contributes to the maintenance of H3K27me3 (Rutowicz et al., 2019), although its direct impact during replication needs to be clarified.

##### PRC2 binds several components of the replication machinery

Interaction of PRC2 with components of the replication machinery likely contributes to its retention at the replication fork (Figure 2B). PRC2 interacts with the proliferating cell nuclear antigen (PCNA) protein through CAF-1 in both plants and animals (Jiang and Berger, 2017; Cheng et al., 2019). Loss of CAF-1 induces strong developmental abnormalities including homeotic transformations in Drosophila that are reminiscent of the phenotypic defects observed in mutants lacking PRC2 activity (Anderson et al., 2011), defects in cell identity maintenance in both mouse embryonic stem cells (ESCs) and Arabidopsis meristems (Kaya et al., 2001; Clémot et al., 2018) as well as increased reprogramming abilities in mouse cells (Cheloufi et al., 2015; Cheloufi and Hochedlinger, 2017). In addition, lack of CAF-1 in Drosophila imaginal discs is associated with a massive decrease of H3K27me3 levels (Anderson et al., 2011; Yee et al., 2019), suggesting that CAF-1 activity is required for the inheritance of H3K27me3 *in vivo*.

Moreover, plant-specific interactions between PRC2 subunits and distinct DNA polymerases have been recently reported in Arabidopsis. Thus, loss of interaction between PRC2 and EARLY IN SHORT DAYS (ESD7), the catalytic subunit of DNA polymerase epsilon (Pol-ε), results in the misexpression of major flowering time regulators such as *AGAMOUS* (*AG*), *SUPPRESSOR OF OVEREXPRESSION OF CO 1* (*SOC1*), and *FLOWERING LOCUS T* (*FT*) (del Olmo et al., 2010, 2016), whose expression is controlled by PRC2. Loss of other DNA polymerases including Pol-α INCURVATA2 (ICU2) and Pol-δ POLD2 also impacts on H3K27me3 distribution, though a direct link with PRC2 subunits remains to be demonstrated (Pedroza-Garcia et al., 2019). These results provide evidence that interactions of PRC2 with multiple DNA polymerases acting at the replication fork likely play a significant role in the maintenance of H3K27me3 landscapes in plants.

The plant-specific protein LIKE HETEROCHROMATIN PROTEIN1 (LHP1) is required for the maintenance of H3K27me3 levels in dividing cells via its interaction with the PRC2 subunit MULTICOPY SUPPRESSOR OF IRA 1 (MSI1), a plant homolog of Nurf55 (Derkacheva et al., 2013; Veluchamy et al., 2016; Feng and Lu, 2017) and is involved in H3K27me3 spreading (Yang et al., 2017). LHP1 also binds several components of the replication fork such as CAF-1 (Li and Luan, 2011; Jiang and Berger, 2017), the CTF4 homolog ENHANCER OF LHP1 (EOL1) (Zhou et al., 2017b), and possibly ICU2 (Barrero et al., 2007; Hyun et al., 2013). Hence, by providing an interaction platform for PRC2 at the replication fork, LHP1 might strengthen the coupling between parental histones recycling and H3K27me3 spreading into nascent chromatin.

Furthermore, LHP1 interacts with components of the plant PRC1 that catalyzes H2A monoubiquitylation (Mozgova and Hennig, 2015). Given its conserved interplay with PRC2 (Yu et al., 2019), PRC1 could be involved in the maintenance of H3K27me3 patterns during replication. Indeed, *in vitro* studies showed that Drosophila PRC1 components remain bound to chromatin during replication (Francis et al., 2009) and that H2A monoubiquitinylation stimulates human PRC2 activity through AEBP2, a mammalian accessory PRC2 subunit (Kalb et al., 2014). In plants, absence of the PRC1 components RING1A/B causes a reduction in H3K27me3 levels over some PRC2 targets (Zhou et al., 2017a). These data indicate therefore that PRC1 could participate, via LHP1, in the recruitment of PRC2 and stimulate its activity at the replication fork.

Taken together, the aforementioned observations suggest that PRC2 is locally retained at the replication fork through multiple and dynamic interactions with components of the replication fork. These synergistic actions likely facilitate the immediate spreading of H3K27me3 over nascent chromatin, thereby participating in the inheritance of transcriptional repression, possibly in a locus-dependent manner (Figure 2B).

#### The Self-Propagation Model: Inherited Parental H3K27me3 Instructs Its Own Spreading Onto Nascent Chromatin

The multimeric structure of PRC2 confers two complementary activities to the complex that are essential for the self-perpetuation of H3K27me3 patterns. Indeed, in addition to the H3K27 trimethylation (*write*) function, PRC2 is also able to *read* H3K27me3 via the specific binding of the ESC subunit (Hansen et al., 2008; Margueron et al., 2009). Moreover, Su(z)12 that is not directly required for H3K27me3 binding, significantly enhances PRC2 affinity for H3K27me3 (Hansen et al., 2008). These built-in *writing* and *reading* properties of PRC2 are not independent from each other since the latter enhances the catalytic activity of the former. Indeed, H3K27me3 binding induces a conformational change of ESC that allosterically stimulates the catalytic activity of E(z) (Margueron et al., 2009). This type of positive feedback loop between chromatin *readers* and *writers* is proposed to be the hallmark property of self-sustained epigenetic memory systems (Reinberg and Vales, 2018). The ability to propagate H3K27me3 over large chromatin domains could provide robustness to PRC2-mediated silencing by limiting the effect of the dilution of H3K27me3 and preventing transcriptional reactivation after replication (Xu et al., 2012). It might also compensate for the stochasticity of parental histone distribution to nascent chromatin, thereby resulting in equal spreading of the mark over daughter strands (Ramachandran and Henikoff, 2015). Thus, faithful restoration of H3K27me3 domains after replication would largely rely on the recycling of parental H3K27me3-containing nucleosomes to nascent chromatin that, in turn, instruct the recruitment of PRC2 and stimulate its *write-and-read* property, leading to the propagation of the mark (Figure 2C, top).

The mechanisms that spatially limit H3K27me3 spreading to preserve the boundaries of H3K27me3 regions through DNA replication are still poorly understood (Yu et al., 2019). H3K27me3 demethylases could be involved in this process, such as the Arabidopsis Jumonji-type EARLY FLOWERING 6 (ELF6), RELATIVE OF EARLY FLOWERING 6 (REF6), and JM13 that restricts H3K27me3 domains in a tissue-specific manner (Yan et al., 2018) or the mammalian KDM6A/UTX that clears up H3K27me3 from tissue-specific enhancers (Saxena et al., 2017). Whether these activities directly contribute to the faithful restoration of PRC2-mediated regulation upon replication remains to be directly investigated.

#### A Change of Paradigm? the Genomic Bookmarking Hypothesis

The prevalence of the self-propagation model described above has been recently challenged by *in vivo* studies suggesting that the inheritance of parental H3K27me3 is not always sufficient to perpetuate long-term transcriptional repression.

In Drosophila, polycomb response elements (PRE) are *cis*-regulatory sequences that locally recruit PRC2 and are necessary and sufficient to mediate long-term silencing (Schuettengruber et al., 2017). Whereas the function of PREs was initially associated with the nucleation of PRC2 to its targets, recent results suggest that these *cis*-elements might also be required for the spreading of H3K27me3 in nascent chromatin and the maintenance of transcriptional silencing through multiple generations of cells. Indeed, excision of a PRE responsible for the repression of the nearby gene resulted in the dilution of H3K27me3 around the excision site and in its transcriptional reactivation within few cells divisions (Coleman and Struhl, 2017; Laprell et al., 2017). However, this decrease in H3K27me3 levels was less important than expected by passive dilution, indicating that PRC2 still maintains a roaming, PRE-independent activity (Coleman and Struhl, 2017). These results argue in favor of a model in which long-term transcriptional silencing also relies on *cis*-regulatory elements that act as bookmarks to anchor PRC2 at silent loci (Figure 2C, bottom). Interestingly, modeling and simulations of H3K27me3 genomic distribution in Drosophila embryos based on this bookmarking model showed that PRE excision, rather than DNA replication, can destabilize H3K27me3 domains (Michieletto et al., 2018). Understanding how many genes rely on this recruiting mechanism and at which step of the replication process such *cis*-regulatory elements come into play to maintain the silenced status across cell division will require further investigations.

In contrast to Drosophila, few PRE-like *cis*-regulatory elements have been characterized in plants (Berger et al., 2011; Förderer et al., 2016; Xiao et al., 2017; Zhou et al., 2018) and mammals (Woo et al., 2010; Schorderet et al., 2013). Furthermore, nucleosome-free and hypomethylated CpG islands in gene regulatory regions were found to act as PRC2 recruitment sites in mammals (Mendenhall et al., 2010; Lynch et al., 2012; Klose et al., 2013; Riising et al., 2014; Højfeldt et al., 2018; Oksuz et al., 2018). *In vivo* and *in vitro* deletion of such PRC2 recruitment sites within the *HoxD* cluster and other loci did not lead to a reduction of H3K27me3 levels around the deletion sites (Schorderet et al., 2013; Oksuz et al., 2018), suggesting that neighboring H3K27me3 levels may be sufficient to recruit PRC2 and maintain the mark across the cluster. However, the kinetics of H3K27me3 deposition in mouse ESCs (mESCs) lacking a PRC2 recruitment site was slowed down compared to the wild-type situation (Oksuz et al., 2018). Similar excision experiments at endogenous loci in Arabidopsis would help determining whether such *cis*-elements also impact on the maintenance of gene repression via PRC2 in plants.

As in Drosophila studies, recent observations in mESCs mitigate the prevalent role of H3K27me3 in recruiting PRC2 at its genomics targets. Indeed, H3K27me3 patterns were accurately re-established via *de novo* methylation by PRC2 in mESCs in which H3K27me3 was totally erased from the whole genome, suggesting that additional cues are sufficient to recruit PRC2 to chromatin independently from H3K27me3 (Højfeldt et al., 2018; Figure 2C, bottom). Whether such cues are *cis*-encoded or result from unidentified factors associated with initial H3K27me3 deposition remains to be determined. For instance, short non-coding RNA transcribed from CpG islands at genes repressed independently of PRC2 have been demonstrated to dynamically recruit PRC2 via Su(z)12, thus participating to silencing maintenance (Kanhere et al., 2010).

The involvement of *cis-*encoded elements in conveying epigenetic memory is also suggested by observations in plant and yeast. During the vernalization process, the *FLOWERING LOCUS C* (*FLC*) gene gradually acquires H3K27me3 in a small domain of approximately three nucleosomes via *de novo* PRC2 recruitment. In a second phase, H3K27me3 then spreads across the whole locus in an *LHP1-*dependent manner to ensure long-term repression. In the *lhp1* mutant, although spreading is affected, H3K27me3 deposition at the nucleation site is maintained across mitoses much longer than predicted if inheritance was ensured exclusively by the stochastic redistribution of parental histones (Yang et al., 2017). Epigenetic memory independent of histone inheritance has also been reported for small heterochromatin domains in yeast, the silenced state of which is inherited at a higher rate than predicted (Saxton and Rine, 2019).

Taken together, these recent advances indicate that the recruitment of PRC2 to nascent chromatin is likely to be even more multifactorial than initially proposed (Margueron and Reinberg, 2011), involving not only self-propagation mechanisms but also H3K27me3-independent cues such as *cis*-regulatory elements and DNA-binding factors (Figure 2). As suggested by the differences observed between drosophila, mammals, and plants, these cues are likely to be both locus- and organism-dependent. These differences may reflect evolutionary changes in fine-tuning the mitotic inheritance of H3K27me3-mediated transcriptional silencing in order to meet distinct developmental strategies.

## Role of H3K27me3 Inheritance in Cell Fate Decisions and Developmental Transitions

Faithful maintenance of H3K27me3 landscape provides a robust memory system that contributes to safeguard the stability of gene expression patterns, hence cell identity, through multiple generations. However, the coordinated and specific changes of transcriptional programs that drive cell fate acquisition entail some flexibility in this PRC2-based memory. Alterations of H3K27me3 deposition at few critical genomic loci, such as those encoding developmental regulators, can be sufficient to trigger major transcriptional changes, as recently exemplified for stomata differentiation in Arabidopsis (Lee et al., 2019). Whereas the release from PRC2 silencing can be achieved by multiple means in interphasic cells including the antagonist action of Trithorax group proteins as well as DNA-binding factors (Brand et al., 2019), recent evidence suggests that local interruption or interference with the mechanisms underlying H3K27me3 restoration during replication could be part of the differentiation process.

### Time to Forget: How Chromatin Replication Might Enable Genes to Escape From PRC2-Mediated Silencing?

Mathematical modeling suggests that transient dilution of H3K27me3 during chromatin replication weakens the stability of silent chromatin by enhancing fast-switching bistability between the silent and active states (Sneppen and Ringrose, 2019). Whereas dilution of H3K27me3 during chromatin replication is likely to promote this instability, additional mechanisms are necessary to counteract the maintenance activities described in the previous section and potentiate the escape from PRC2 repression. Conceptually, release of PRC2 repression during replication could be achieved at a given locus through distinct, non-mutually exclusive mechanisms that would locally prevent the transfer of H3 parental histones into nascent chromatin, bias the distribution of parental H3K27me3 into daughter strands or hinder PRC2 recruitment or activity over nascent chromatin.

#### Biasing H3K27me3 Inheritance to Break the Symmetry Between Daughter Chromatins?

Recent work in *Drosophila* male germline stem cells showed that parental H3 can be locally re-deposited in a preferential manner to the leading strand during DNA replication, suggesting that asymmetric division can arise from asymmetric inheritance of parental histones (Wooten et al., 2019). Although the factors responsible for this asymmetric deposition of parental H3 remain to be identified, regulation of asymmetric division could rely on factors disrupting specifically one of the strand-specific recycling pathways of parental H3 (Gan et al., 2018; Petryk et al., 2018; Yu et al., 2018; Figures 1B,B′). How such a mechanism affects the inheritance of H3K27me3-marked parental histones and whether it directly participates in asymmetric cell fate decisions requires further investigations in animals and plants.

#### Modulation of Replication-Dependent H3K27me3 Inheritance Enables Identity Switches

Restoration kinetics of pre-replication H3K27me3 levels may be critical to set out a window of opportunity during which a locus can escape from PRC2-mediated silencing. Interestingly, the kinetics of H3K27me3 restoration seem to differ between plant and animal cells. In mammalian cells, H3K27me3 levels are not fully restored before the late post-mitotic G1 phase (Xu et al., 2012; Alabert et al., 2015). Such a slow methylation rate has been proposed to filter out fluctuations of transcription factors, thus ensuring the stability of silent states through cell divisions (Berry et al., 2017). In tobacco BY-2 cells though, H3K27me3 levels are restored as soon as early pre-mitotic G2 phase. This suggests that plant cells have evolved specific mechanisms allowing for rapid restoration of H3K27me3 after DNA replication (Jiang and Berger, 2017). In contrast to animal cells in which PRC2 is the only complex catalyzing mono-, di-, and tri-methylation of H3K27, in plant cells a pathway involving the plant-specific H3K27 mono-methylases ATXR5/6 exist, independently from PRC2 (Jacob et al., 2014). Anchoring of both ATXR5/6 and PRC2 at the replication fork might favor a rapid restoration of H3K27me3 after replication via the deposition of H3K27me1 on newly synthesized nucleosome, which could serve as a template for PRC2 (Jiang and Berger, 2017). However, understanding the extent to which the kinetics of H3K27me3 restoration impacts on the ability of cells to perpetuate PRC2-driven transcriptional memory requires further studies.

Modulation of H3K27me3 restoration kinetics has been associated with cell fate decisions in mammalian cells. For instance, induction of mESC differentiation slows down the restoration of H3K27me3 due to the recruitment of the H3K27me3 demethylase, ubiquitously transcribed tetratricopeptide repeat X chromosome (UTX) downstream of the replication fork, thereby preventing the spreading of H3K27me3 (Petruk et al., 2017). Slowing down the restoration of H3K27me3 might enlarge the window of opportunity during which chromatin is in a state permissive for transcriptional reprogramming, allowing for transcription factors to bind and activate their target genes. In agreement with this, computational simulations suggest that H3K27me3 demethylase activity during replication does favor bistability switching to the active state (Sneppen and Ringrose, 2019). Determining whether the EFL6/REF6/JMJ13 homologs also participate in such replication-coupled transcriptional reprogramming awaits direct investigations.

In addition, differentiation of ESCs is enhanced by ex14D-EZH2, a splicing variant of EZH2 with reduced catalytic activity (Mu et al., 2018). This indicates that the presence of tightly regulated variants of PRC2 may facilitate differentiation by impeding rapid H3K27me3 spreading in nascent chromatin at specific loci.

Finally, the equilibrium between the kinetics of H3K27 trimethylation and the mitotic rate may strongly influence the ability of a cell to perpetuate PRC2-mediated silencing through mitosis. Intuitively, if the cell cycle length is shorter than the time required for full H3K27me3 restoration, then H3K27me3 could be gradually lost within few cell divisions. In Arabidopsis, a coupling between the regulation of cell division timing and replication-dependent dilution of H3K27me3 has been proposed to control the termination of the stem cell pool during floral development (Sun et al., 2014). This coupling fine-tunes the number of floral organs produced by the meristem and thus conditions the reproductive fitness of the plant. Interestingly, this mechanism might be conserved in other dicots such as tomato (Bollier et al., 2018) and monocots such as the rice (Conrad et al., 2014).

### Chromatin Replication Can Potentiate PRC2-Mediated Silencing

Whereas cells can take advantage of chromatin destabilization during the S-phase to unlock the transcription of specific PRC2 target genes, chromatin replication was also reported to potentiate PRC2-mediated silencing. In ESCs, proper establishment of H3K27me3 at pluripotency genes such as Nanog, Oct4, and Sox2 is likely to be critical for pluripotency exit and rely on the local recruitment of PRC2 by CAF1 in S-phase (Cheng et al., 2019).

Whereas earlier studies in Arabidopsis showed that the repression of *FLC* established during the vernalization process is not maintained in post-mitotic cells of mature leaves (Finnegan and Dennis, 2007), replication was recently confirmed to be required for H3K27me3 spreading and the establishment of long-term silencing of *FLC* (Jiang and Berger, 2017; Sharif and Koseki, 2017; Yang et al., 2017). In keeping with this, mathematical modeling revealed that the sharp switch of *FLC* to the silent state is consistent with a replication-dependent spreading of H3K27me3 at the locus, suggesting that the coupling between PRC2-mediated silencing and chromatin replication can generate a quantitative integrator of environmental signals (Angel et al., 2011).

## Conclusion

**FIGURE 3:**
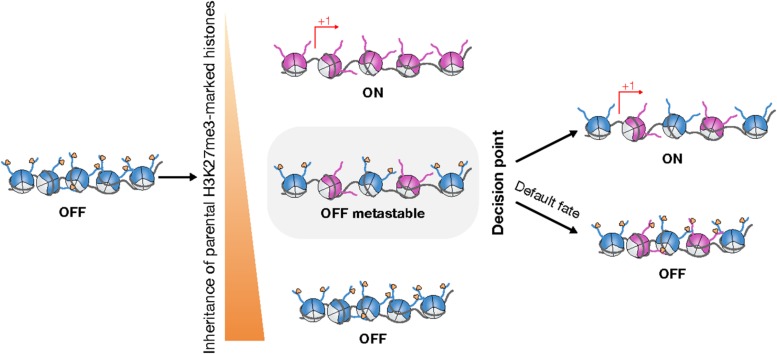
Chromatin replication can potentiate cell fate transitions. During chromatin replication, asymmetric inheritance of parental H3K27me3 can lead to transcriptional reactivation in only one daughter cell **(Middle, Top, Bottom)**. In case of symmetric inheritance of parental H3K27me3, repressive chromatin is switched from a stable repressive state to a metastable state that is permissive for epigenetic reprogramming **(Middle)**. By default, H3K27me3 landscapes are restored such that repressive chromatin remains silent **(Right, Bottom)**. Nevertheless, local disruption of the restoration of repressive chromatin can trigger specific changes in gene expression **(Right, Top)**.

The S-phase is particularly challenging for the maintenance of transcriptional programs, hence for cell identity. Cell identity is safeguarded during chromatin replication by the inheritance of the H3K27me3 landscape, which enables the perpetuation of PRC2-mediated transcriptional repression. H3K27me3 inheritance relies on both the accurate re-deposition of parental H3K27me3-marked H3 (Figure 1) and the spreading of H3K27me3 downstream of the replication fork (Figures 2, 3). Recent advances provide new evidence that cell identity switches during developmental processes are intrinsically coupled with chromatin replication and the regulation of PRC2-mediated silencing (Figure 3). These findings strongly support the hypothesis that the S-phase opens a window of opportunity for transcriptional reprogramming and cell fate decisions.

While our understanding of the molecular mechanisms underlying H3K27me3 inheritance is gradually increasing and points toward overall conservation between organisms, differences have also emerged in plants. Thus, separation of H3K27me1 from H3K27me3 catalysis into different pathways illustrates a plant-specific innovation that might directly impact on the kinetics of H3K27me3 re-establishment during replication. In addition, developmental processes more specific to plant biology, such as continuous growth or widespread endoreplication, could introduce additional differences in the mechanistic and developmental impacts of H3K27me3 inheritance. Further investigation of PRC2 interactions within the micro-environment of the replication fork will provide key insights to understand how H3K27me3 inheritance is modulated at specific genomic loci in order to fine-tune developmental processes in space and time.

## Author Contributions

AH and FR designed the outline of the manuscript with additional input from CJ. All authors contributed to the writing of the manuscript and approved the final manuscript. AH contributed to all figure designs.

## Conflict of Interest

The authors declare that the research was conducted in the absence of any commercial or financial relationships that could be construed as a potential conflict of interest.

## References

[B1] Alabert C. Barth T. K. Reverón-Gómez N. Sidoli S. Schmidt A. Jensen O. N. (2015). Two distinct modes for propagation of histone PTMs across the cell cycle. *Genes Dev.* 29 585–590. 10.1101/gad.256354.114 25792596 PMC4378191

[B2] Alabert C. Bukowski-Wills J.-C. Lee S.-B. Kustatscher G. Nakamura K. de Lima Alves F. (2014). Nascent chromatin capture proteomics determines chromatin dynamics during DNA replication and identifies unknown fork components. *Nat. Cell Biol.* 16 281–293. 10.1038/ncb2918 24561620 PMC4283098

[B3] Anderson A. E. Karandikar U. C. Pepple K. L. Chen Z. Bergmann A. Mardon G. (2011). The enhancer of trithorax and polycomb gene Caf1/p55 is essential for cell survival and patterning in drosophila development. *Dev. Camb. Engl.* 138 1957–1966. 10.1242/dev.058461 21490066 PMC3082301

[B4] Angel A. Song J. Dean C. Howard M. (2011). A Polycomb-based switch underlying quantitative epigenetic memory. *Nature* 476 105–108. 10.1038/nature10241 21785438

[B5] Annunziato A. T. (2015). The fork in the road: histone partitioning during DNA replication. *Genes* 6 353–371. 10.3390/genes6020353 26110314 PMC4488668

[B6] Barrero J. M. González-Bayón R. del Pozo J. C. Ponce M. R. Micol J. L. (2007). INCURVATA2 encodes the catalytic subunit of DNA polymerase α and interacts with genes involved in chromatin-mediated cellular memory in *Arabidopsis thaliana*. *Plant Cell* 19 2822–2838. 10.1105/tpc.107.054130 17873092 PMC2048701

[B7] Benoit M. Simon L. Desset S. Duc C. Cotterell S. Poulet A. (2019). Replication-coupled histone H3.1 deposition determines nucleosome composition and heterochromatin dynamics during *Arabidopsis* seedling development. *New Phytol.* 221 385–398. 10.1111/nph.15248 29897636

[B8] Berger N. Dubreucq B. Roudier F. Dubos C. Lepiniec L. (2011). Transcriptional regulation of Arabidopsis leafy cotyledon2 involves RLE, a cis-Element that regulates trimethylation of histone H3 at Lysine-27[W]. *Plant Cell* 23 4065–4078. 10.1105/tpc.111.087866 22080598 PMC3246333

[B9] Berry S. Dean C. Howard M. (2017). Slow chromatin dynamics allow polycomb target genes to filter fluctuations in transcription factor activity. *Cell Syst.* 4 445–457.e8. 10.1016/j.cels.2017.02.013 28342717 PMC5409831

[B10] Bollier N. Sicard A. Leblond J. Latrasse D. Gonzalez N. Gévaudant F. (2018). At-MINI ZINC FINGER2 and Sl-INHIBITOR OF MERISTEM ACTIVITY, a conserved missing link in the regulation of floral meristem termination in *Arabidopsis* and Tomato. *Plant Cell* 30 83–100. 10.1105/tpc.17.00653 29298836 PMC5810569

[B11] Brand M. Nakka K. Zhu J. Dilworth F. J. (2019). Polycomb/Trithorax antagonism: cellular memory in stem cell fate and function. *Cell Stem Cell* 24 518–533. 10.1016/j.stem.2019.03.005 30951661 PMC6866673

[B12] Cheloufi S. Elling U. Hopfgartner B. Jung Y. L. Murn J. Ninova M. (2015). The histone chaperone CAF-1 safeguards somatic cell identity. *Nature* 528 218–224. 10.1038/nature15749 26659182 PMC4866648

[B13] Cheloufi S. Hochedlinger K. (2017). Emerging roles of the histone chaperone CAF-1 in cellular plasticity. *Curr. Opin. Genet. Dev.* 46 83–94. 10.1016/j.gde.2017.06.004 28692904 PMC5813839

[B14] Cheng L. Zhang X. Wang Y. Gan H. Xu X. Lv X. (2019). Chromatin assembly factor 1 (CAF-1) facilitates the establishment of facultative heterochromatin during pluripotency exit. *Nucleic Acids Res.* 47 11114–11131. 10.1093/nar/gkz858 31586391 PMC6868363

[B15] Clémot M. Molla-Herman A. Mathieu J. Huynh J.-R. Dostatni N. (2018). The replicative histone chaperone CAF1 is essential for the maintenance of identity and genome integrity in adult stem cells. *Dev. Camb. Engl.* 145:dev161190. 10.1242/dev.161190 30093554

[B16] Coleman R. T. Struhl G. (2017). Causal role for inheritance of H3K27me3 in maintaining the OFF state of a drosophila HOX gene. *Science* 356:eaai8236. 10.1126/science.aai8236 28302795 PMC5595140

[B17] Conrad L. J. Khanday I. Johnson C. Guiderdoni E. An G. Vijayraghavan U. (2014). The polycomb group gene EMF2B is essential for maintenance of floral meristem determinacy in rice. *Plant J.* 80 883–894. 10.1111/tpj.12688 25279942

[B18] del Olmo I. López J. A. Vázquez J. Raynaud C. Piñeiro M. Jarillo J. A. (2016). Arabidopsis DNA polymerase ϵ recruits components of Polycomb repressor complex to mediate epigenetic gene silencing. *Nucleic Acids Res.* 44 5597–5614. 10.1093/nar/gkw156 26980282 PMC4937302

[B19] del Olmo I. López-González L. Martín-Trillo M. M. Martínez-Zapater J. M. Piñeiro M. Jarillo J. A. (2010). EARLY IN SHORT DAYS 7 (ESD7) encodes the catalytic subunit of DNA polymerase epsilon and is required for flowering repression through a mechanism involving epigenetic gene silencing. *Plant J. Cell Mol. Biol.* 61 623–636. 10.1111/j.1365-313X.2009.04093.x 19947980

[B20] Derkacheva M. Steinbach Y. Wildhaber T. Mozgová I. Mahrez W. Nanni P. (2013). Arabidopsis MSI1 connects LHP1 to PRC2 complexes. *EMBO J.* 32 2073–2085. 10.1038/emboj.2013.145 23778966 PMC3715863

[B21] Escobar T. Oksuz O. Descostes N. Bonasio R. Reinberg D. (2018). Precise re-deposition of nucleosomes on repressive chromatin domains sustain epigenetic inheritance during DNA replication. *BioRxiv* [Preprint], 10.1101/418707

[B22] Feng J. Lu J. (2017). LHP1 Could Act as an Activator and a Repressor of Transcription in Plants. *Front. Plant Sci.* 8:2041. 10.3389/fpls.2017.02041 29234344 PMC5712405

[B23] Finnegan E. J. Dennis E. S. (2007). Vernalization-induced trimethylation of histone H3 lysine 27 at FLC is not maintained in mitotically quiescent cells. *Curr. Biol. CB* 17 1978–1983. 10.1016/j.cub.2007.10.026 17980595

[B24] Förderer A. Zhou Y. Turck F. (2016). The age of multiplexity: recruitment and interactions of Polycomb complexes in plants. *Curr. Opin. Plant Biol.* 29 169–178. 10.1016/j.pbi.2015.11.010 26826786

[B25] Francis N. J. Follmer N. E. Simon M. D. Aghia G. Butler J. D. (2009). Polycomb proteins remain bound to chromatin and DNA during DNA replication *in vitro*. *Cell* 137 110–122. 10.1016/j.cell.2009.02.017 19303136 PMC2667909

[B26] Gan H. Serra-Cardona A. Hua X. Zhou H. Labib K. Yu C. (2018). The Mcm2-Ctf4-Polα axis facilitates parental histone H3-H4 transfer to lagging strands. *Mol. Cell* 72 140.e–151.e. 10.1016/j.molcel.2018.09.001 30244834 PMC6193272

[B27] Hammond C. M. Strømme C. B. Huang H. Patel D. J. Groth A. (2017). Histone chaperone networks shaping chromatin function. *Nat. Rev. Mol. Cell Biol.* 18 141–158. 10.1038/nrm.2016.159 28053344 PMC5319910

[B28] Hansen K. H. Bracken A. P. Pasini D. Dietrich N. Gehani S. S. Monrad A. (2008). A model for transmission of the H3K27me3 epigenetic mark. *Nat. Cell Biol.* 10 1291–1300. 10.1038/ncb1787 18931660

[B29] He H. Li Y. Dong Q. Chang A.-Y. Gao F. Chi Z. (2017). Coordinated regulation of heterochromatin inheritance by Dpb3–Dpb4 complex. *Proc. Natl. Acad. Sci.* 114 12524–12529. 10.1073/pnas.1712961114 29109278 PMC5703312

[B30] Højfeldt J. W. Laugesen A. Willumsen B. M. Damhofer H. Hedehus L. Tvardovskiy A. (2018). Accurate H3K27 methylation can be established de novo by SUZ12-directed PRC2. *Nat. Struct. Mol. Biol.* 25 225–232. 10.1038/s41594-018-0036-6 29483650 PMC5842896

[B31] Huang H. Strømme C. B. Saredi G. Hödl M. Strandsby A. González-Aguilera C. (2015). A unique binding mode enables MCM2 to chaperone histones H3–H4 at replication forks. *Nat. Struct. Mol. Biol.* 22 618–626. 10.1038/nsmb.3055 26167883 PMC4685956

[B32] Hyun Y. Yun H. Park K. Ohr H. Lee O. Kim D.-H. (2013). The catalytic subunit of *Arabidopsis* DNA polymerase α ensures stable maintenance of histone modification. *Dev. Camb. Engl.* 140 156–166. 10.1242/dev.084624 23154417

[B33] Jacob Y. Bergamin E. Donoghue M. T. A. Mongeon V. LeBlanc C. Voigt P. (2014). Selective methylation of histone H3 variant H3.1 regulates heterochromatin replication. *Science* 343 1249–1253. 10.1126/science.1248357 24626927 PMC4049228

[B34] Jiang D. Berger F. (2017). DNA replication–coupled histone modification maintains Polycomb gene silencing in plants. *Science* 357 1146–1149. 10.1126/science.aan4965 28818970

[B35] Kalb R. Latwiel S. Baymaz H. I. Jansen P. W. T. C. Müller C. W. Vermeulen M. (2014). Histone H2A monoubiquitination promotes histone H3 methylation in Polycomb repression. *Nat. Struct. Mol. Biol.* 21 569–571. 10.1038/nsmb.2833 24837194

[B36] Kanhere A. Viiri K. Araújo C. C. Rasaiyaah J. Bouwman R. D. Whyte W. A. (2010). Short RNAs are transcribed from repressed polycomb target genes and interact with polycomb repressive complex-2. *Mol. Cell* 38 675–688. 10.1016/j.molcel.2010.03.019 20542000 PMC2886029

[B37] Kaya H. Shibahara K. I. Taoka K. I. Iwabuchi M. Stillman B. Araki T. (2001). FASCIATA genes for chromatin assembly factor-1 in arabidopsis maintain the cellular organization of apical meristems. *Cell* 104 131–142. 10.1016/s0092-8674(01)00197-0 11163246

[B38] Klose R. J. Cooper S. Farcas A. M. Blackledge N. P. Brockdorff N. (2013). Chromatin sampling–an emerging perspective on targeting polycomb repressor proteins. *PLoS Genet.* 9:e1003717. 10.1371/journal.pgen.1003717 23990804 PMC3749931

[B39] Laprell F. Finkl K. Müller J. (2017). Propagation of Polycomb-repressed chromatin requires sequence-specific recruitment to DNA. *Science* 356 85–88. 10.1126/science.aai8266 28302792

[B40] Lee L. R. Wengier D. L. Bergmann D. C. (2019). Cell-type–specific transcriptome and histone modification dynamics during cellular reprogramming in the *Arabidopsis* stomatal lineage. *Proc. Natl. Acad. Sci. U.S.A.* 116 21914–21924. 10.1073/pnas.1911400116 31594845 PMC6815143

[B41] Li H. Luan S. (2011). The cyclophilin AtCYP71 interacts with CAF-1 and LHP1 and functions in multiple chromatin remodeling processes. *Mol. Plant* 4 748–758. 10.1093/mp/ssr036 21596687

[B42] Lynch M. D. Smith A. J. H. De Gobbi M. Flenley M. Hughes J. R. Vernimmen D. (2012). An interspecies analysis reveals a key role for unmethylated CpG dinucleotides in vertebrate Polycomb complex recruitment. *EMBO J.* 31 317–329. 10.1038/emboj.2011.399 22056776 PMC3261560

[B43] Margueron R. Justin N. Ohno K. Sharpe M. L. Son J. Drury W. J. (2009). Role of the polycomb protein EED in the propagation of repressive histone marks. *Nature* 461 762–767. 10.1038/nature08398 19767730 PMC3772642

[B44] Margueron R. Reinberg D. (2011). The Polycomb Complex PRC2 and its Mark in Life. *Nature* 469 343–349. 10.1038/nature09784 21248841 PMC3760771

[B45] Masai H. Foiani M. (2018). *DNA Replication: From Old Principles to New Discoveries.* Berlin: Springer.

[B46] Mendenhall E. M. Koche R. P. Truong T. Zhou V. W. Issac B. Chi A. S. (2010). GC-rich sequence elements recruit PRC2 in mammalian ES cells. *PLoS Genet.* 6:e1001244. 10.1371/journal.pgen.1001244 21170310 PMC3000368

[B47] Michieletto D. Chiang M. Colì D. Papantonis A. Orlandini E. Cook P. R. (2018). Shaping epigenetic memory via genomic bookmarking. *Nucleic Acids Res.* 46 83–93. 10.1093/nar/gkx1200 29190361 PMC5758908

[B48] Mikulski P. Hohenstatt M. L. Farrona S. Smaczniak C. Stahl Y. Kalyanikrishna. (2019). The chromatin-associated protein PWO1 interacts with plant nuclear lamin-like components to regulate nuclear size. *Plant Cell* 31 1141–1154. 10.1105/tpc.18.00663 30914470 PMC6533023

[B49] Mozgova I. Hennig L. (2015). The polycomb group protein regulatory network. *Annu. Rev. Plant Biol.* 66 269–296. 10.1146/annurev-arplant-043014-115627 25621513

[B50] Mu W. Starmer J. Yee D. Magnuson T. (2018). EZH2 variants differentially regulate polycomb repressive complex 2 in histone methylation and cell differentiation. *Epigenet. Chromat.* 11:71. 10.1186/s13072-018-0242-9 30522506 PMC6282306

[B51] Oksuz O. Narendra V. Lee C.-H. Descostes N. LeRoy G. Raviram R. (2018). Capturing the onset of PRC2-mediated repressive domain formation. *Mol. Cell* 70 1149–1162.e5. 10.1016/j.molcel.2018.05.023 29932905 PMC7700016

[B52] Pedroza-Garcia J.-A. De Veylder L. Raynaud C. (2019). Plant DNA Polymerases. *Int. J. Mol. Sci.* 20:4814. 10.3390/ijms20194814 31569730 PMC6801657

[B53] Petruk S. Cai J. Sussman R. Sun G. Kovermann S. K. Mariani S. A. (2017). Delayed accumulation of H3K27me3 on nascent DNA is essential for recruitment of transcription factors at early stages of stem cell differentiation. *Mol. Cell* 66 247–257.e5. 10.1016/j.molcel.2017.03.006 28410996 PMC5412717

[B54] Petryk N. Dalby M. Wenger A. Stromme C. B. Strandsby A. Andersson R. (2018). MCM2 promotes symmetric inheritance of modified histones during DNA replication. *Science* 361 1389–1392. 10.1126/science.aau0294 30115746

[B55] Ramachandran S. Ahmad K. Henikoff S. (2017). Capitalizing on disaster: establishing chromatin specificity behind the replication fork. *Bioessays* 39:1600150. 10.1002/bies.201600150 28133760 PMC5513704

[B56] Ramachandran S. Henikoff S. (2015). Replicating nucleosomes. *Sci. Adv.* 1:e1500587. 10.1126/sciadv.1500587 26269799 PMC4530793

[B57] Reinberg D. Vales L. D. (2018). Chromatin domains rich in inheritance. *Science* 361 33–34. 10.1126/science.aat7871 29976815

[B58] Reverón-Gómez N. González-Aguilera C. Stewart-Morgan K. R. Petryk N. Flury V. Graziano S. (2018). Accurate recycling of parental histones reproduces the histone modification landscape during DNA replication. *Mol. Cell.* 72 239–249.e5. 10.1016/j.molcel.2018.08.010 30146316 PMC6202308

[B59] Riising E. M. Comet I. Leblanc B. Wu X. Johansen J. V. Helin K. (2014). Gene silencing triggers polycomb repressive complex 2 recruitment to CpG islands genome wide. *Mol. Cell* 55 347–360. 10.1016/j.molcel.2014.06.005 24999238

[B60] Rutowicz K. Lirski M. Mermaz B. Teano G. Schubert J. Mestiri I. (2019). Linker histones are fine-scale chromatin architects modulating developmental decisions in *Arabidopsis*. *Genome Biol.* 20:157. 10.1186/s13059-019-1767-3 31391082 PMC6685187

[B61] Saxena M. Roman A. K. S. O’Neill N. K. Sulahian R. Jadhav U. Shivdasani R. A. (2017). Transcription factor-dependent “anti-repressive” mammalian enhancers exclude H3K27me3 from extended genomic domains. *Genes Dev.* 31 2391–2404. 10.1101/gad.308536.117 29321178 PMC5795785

[B62] Saxton D. S. Rine J. (2019). Epigenetic memory independent of symmetric histone inheritance. *eLife* 8:e51421. 10.7554/eLife.51421 31613222 PMC6850775

[B63] Schorderet P. Lonfat N. Darbellay F. Tschopp P. Gitto S. Soshnikova N. (2013). A genetic approach to the recruitment of PRC2 at the HoxD Locus. *PLoS Genet.* 9:e1003951. 10.1371/journal.pgen.1003951 24244202 PMC3820793

[B64] Schuettengruber B. Bourbon H.-M. Croce L. D. Cavalli G. (2017). Genome regulation by polycomb and trithorax: 70 Years and counting. *Cell* 171 34–57. 10.1016/j.cell.2017.08.002 28938122

[B65] Serra-Cardona A. Zhang Z. (2018). Replication-coupled nucleosome assembly in the passage of epigenetic information and cell identity. *Trends Biochem. Sci.* 43 136–148. 10.1016/j.tibs.2017.12.003 29292063 PMC5805396

[B66] Sharif J. Koseki H. (2017). No winter lasts forever: polycomb complexes convert epigenetic memory of cold into flowering. *Dev. Cell* 42 563–564. 10.1016/j.devcel.2017.09.004 28950098

[B67] Sharif J. Koseki H. (2018). Rewriting the past: de novo activity of PRC2 restores global H3K27 methylation patterns. *Nat. Struct. Mol. Biol.* 25:197 10.1038/s41594-018-0039-33 29507402

[B68] Snedeker J. Wooten M. Chen X. (2017). The inherent asymmetry of DNA replication. *Annu. Rev. Cell Dev. Biol.* 33 291–318. 10.1146/annurev-cellbio-100616-060447 28800257 PMC5695668

[B69] Sneppen K. Ringrose L. (2019). Theoretical analysis of Polycomb-Trithorax systems predicts that poised chromatin is bistable and not bivalent. *Nat. Commun.* 10 1–18. 10.1038/s41467-019-10130-2 31086177 PMC6513952

[B70] Sun B. Looi L.-S. Guo S. He Z. Gan E.-S. Huang J. (2014). Timing Mechanism Dependent on Cell Division Is Invoked by Polycomb Eviction in Plant Stem Cells. *Science* 343:1248559. 10.1126/science.1248559 24482483

[B71] Teves S. S. Henikoff S. (2014). DNA torsion as a feedback mediator of transcription and chromatin dynamics. *Nucleus* 5 211–218. 10.4161/nucl.29086 24819949 PMC4133216

[B72] Veluchamy A. Jégu T. Ariel F. Latrasse D. Mariappan K. G. Kim S.-K. (2016). LHP1 regulates H3K27me3 spreading and shapes the three-dimensional conformation of the *Arabidopsis* genome. *PLoS One* 11:e0158936. 10.1371/journal.pone.0158936 27410265 PMC4943711

[B73] Woo C. J. Kharchenko P. V. Daheron L. Park P. J. Kingston R. E. (2010). A region of the human HOXD cluster that confers polycomb-group responsiveness. *Cell* 140 99–110. 10.1016/j.cell.2009.12.022 20085705 PMC3324942

[B74] Wooten M. Snedeker J. Nizami Z. F. Yang X. Ranjan R. Urban E. (2019). Asymmetric histone inheritance via strand-specific incorporation and biased replication fork movement. *Nat. Struct. Mol. Biol.* 26 732–743. 10.1038/s41594-019-0269-z 31358945 PMC6684448

[B75] Xiao J. Jin R. Wagner D. (2017). Developmental transitions: integrating environmental cues with hormonal signaling in the chromatin landscape in plants. *Genome Biol.* 18:88. 10.1186/s13059-017-1228-9 28490341 PMC5425979

[B76] Xu M. Wang W. Chen S. Zhu B. (2012). A model for mitotic inheritance of histone lysine methylation. *EMBO Rep.* 13 60–67. 10.1038/embor.2011.206 22056817 PMC3246248

[B77] Yan W. Chen D. Smaczniak C. Engelhorn J. Liu H. Yang W. (2018). Dynamic and spatial restriction of Polycomb activity by plant histone demethylases. *Nat. Plants* 4:681. 10.1038/s41477-018-0219-5 30104650

[B78] Yang H. Berry S. Olsson T. S. G. Hartley M. Howard M. Dean C. (2017). Distinct phases of Polycomb silencing to hold epigenetic memory of cold in *Arabidopsis*. *Science* 357 1142–1145. 10.1126/science.aan1121 28818969

[B79] Yee W. B. Delaney P. M. Vanderzalm P. J. Ramachandran S. Fehon R. G. (2019). The CAF-1 complex couples hippo pathway target gene expression and DNA replication. *Mol. Biol. Cell* 30 2929–2942. 10.1091/mbc.E19-07-0387 31553691 PMC6822585

[B80] Yu C. Gan H. Serra-Cardona A. Zhang L. Gan S. Sharma S. (2018). A mechanism for preventing asymmetric histone segregation onto replicating DNA strands. *Science* 361 1386–1389. 10.1126/science.aat8849 30115745 PMC6597248

[B81] Yu J.-R. Lee C.-H. Oksuz O. Stafford J. M. Reinberg D. (2019). PRC2 is high maintenance. *Genes Dev.* 33 903–935. 10.1101/gad.325050.119 31123062 PMC6672058

[B82] Zhou Y. Romero-Campero F. J. Gómez-Zambrano Á Turck F. Calonje M. (2017a). H2A monoubiquitination in *Arabidopsis thaliana* is generally independent of LHP1 and PRC2 activity. *Genome Biol* 18:69. 10.1186/s13059-017-1197-z 28403905 PMC5389094

[B83] Zhou Y. Tergemina E. Cui H. Förderer A. Hartwig B. Velikkakam James G. (2017b). Ctf4-related protein recruits LHP1-PRC2 to maintain H3K27me3 levels in dividing cells in *Arabidopsis thaliana*. *Proc. Natl. Acad. Sci. U.S.A.* 114 4833–4838. 10.1073/pnas.1620955114 28428341 PMC5422822

[B84] Zhou Y. Wang Y. Krause K. Yang T. Dongus J. A. Zhang Y. (2018). Telobox motifs recruit CLF/SWN-PRC2 for H3K27me3 deposition via TRB factors in *Arabidopsis*. *Nat. Genet.* 50 638–644. 10.1038/s41588-018-0109-9 29700471

